# Sequencing-based large-scale genomics approaches with small numbers of isolated maize meiocytes

**DOI:** 10.3389/fpls.2014.00057

**Published:** 2014-02-25

**Authors:** Stefanie Dukowic-Schulze, Anitha Sundararajan, Thiruvarangan Ramaraj, Joann Mudge, Changbin Chen

**Affiliations:** ^1^Department of Horticultural Science, University of MinnesotaSt. Paul, MN, USA; ^2^National Center for Genome ResourcesSanta Fe, NM, USA

**Keywords:** meiocytes, meiosis, Illumina sequencing, RNA-seq, ChIP-seq, DNA methylation, small RNA, maize

## Abstract

High-throughput sequencing has become the large-scale approach of choice to study global gene expression and the distribution of specific chromatin marks and features. However, the limited availability of large amounts of purified cells made it very challenging to apply sequencing-based techniques in plant meiosis research in the past. In this paper, we describe a method to isolate meiocytes from maize anthers and detailed protocols to successfully perform RNA-seq, smRNA-seq, H3K4me3-ChIP-seq, and DNA bisulfite conversion sequencing with 5000–30,000 isolated maize male meiotic cells. These methods can be adjusted for other flowering plant species as well.

## Introduction

Plant meiosis research has a long and fruitful history, started by Gregor Mendel and his heredity studies in peas even before DNA was discovered (Mendel, 1865; Kemp, 2002). The advancements of modern light microscopy facilitated cytological studies of meiosis in many plant species in the last century, and especially cytogenetics done in maize (Rhoades, 1955) propelled the knowledge of meiosis and meiotic recombination forward. New technologies opened up new venues to explore plant meiosis, with forward genetics using *Arabidopsis* mutant studies being more current and predominant (Mercier and Grelon, 2008). With the rise of high-throughput sequencing technologies, even more possibilities now exist, which will give us more insight into global aspects during meiosis.

In the field of plant meiosis, large-scale transcriptome studies were the first global approaches performed using microarray or sequencing technologies on isolated meiocytes (Chen et al., 2010; Libeau et al., 2011; Yang et al., 2011). Additionally of interest are chromatin features, especially regarding their significance in meiotic recombination. More recently, studies began to map and correlate sites of recombination and chromatin features in plants with high resolution (Giraut et al., 2011; Lu et al., 2012; Choi et al., 2013).

Meiotic recombination can be detected in any somatic plant cells by analyzing crossover events in the progeny of parents with enough sequence divergence. Also, studies such as chromatin immunoprecipitation (ChIP) for meiosis-specific proteins can be performed using whole tissues containing the cells of interest (He et al., 2013) without being overly concerned about possible contamination from non-meiotic cells. However, if a global approach is intended for anything that is present throughout the whole plant, such as histones and housekeeping genes, using pure material is mandatory. Thus, meiocytes should be isolated for any high-resolution experiment exploring the meiotic transcriptome or universal chromatin features like DNA methylation or DNA-associated ubiquitous proteins like histones. Even if most of the latter data exists for plants in general, it should not be assumed that somatic chromatin feature distribution is universally the same. If data from whole anthers or any somatic tissue are used due to limited availability of data from isolated meiocytes, one should be cautious to correlate it with meiocyte data and to make statements for meiosis-specific events.

Unfortunately, the isolation of plant meiocytes is neither easy to perform nor does it yield a very high amount of cells within a reasonable time. Schmidt et al. (2012) nicely laid out possible methods that exist for isolating meiocytes, including laser-assisted microdissection (LAM, Becker et al., 1997), isolation of nuclei tagged in specific cell types (INTACT, Deal and Henikoff, 2011), gradient centrifugation in later stages, and microcapillary collection. The last method has independently been used for analysis of the *Arabidopsis* meiotic transcriptome by different laboratories and seems to be the most promising approach (Chen et al., 2010; Yang et al., 2011; Schmidt et al., 2012; Chen and Retzel, 2013).

**Figure 1:**
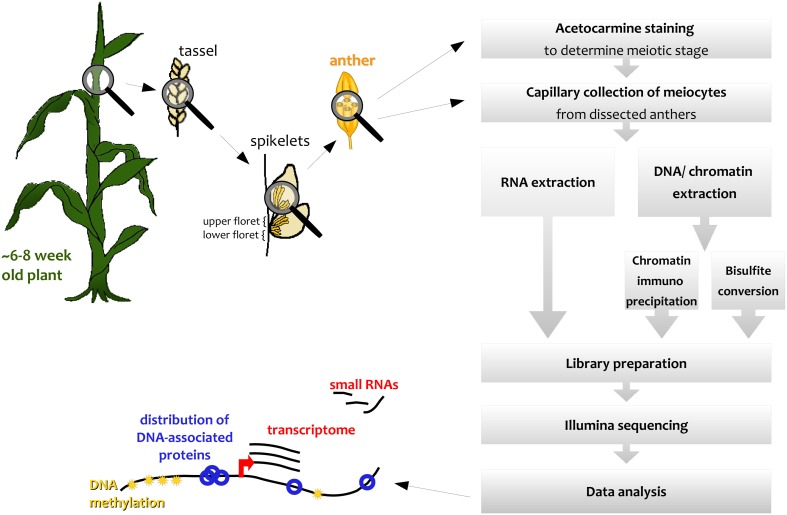
**Simplified workflow overview**.

We describe here how this capillary collection of meiocytes (CCM) and diverse large-scale downstream analyses can be applied to maize (Figure 1); however, CCM is not limited to the two model organisms *Arabidopsis* and maize, and can also be adapted for other species, as we did, for example, in rice. The number of required collected meiocytes depends on the downstream analysis, ranging from 5000 maize meiocytes for RNA-seq to 20,000 or more for ChIP-seq of chromatin-bound proteins.

## Materials and methods

### Plant material preparation

Maize plants (*Zea mays*, different inbred lines) were grown in the greenhouse, 16 h light (at least 450 μM × m^−2^ × s^−1^) at 24°C, 8 h dark at 22°C. One pre-germinated seedling per pot (diameter ~30 cm, fill height ~20–25 cm) was planted in a 2:1 mix of field top soil and SunGro LC8 (Sun Gro Horticulture, Agawam, MA, US). Sand was layered on top to prevent most weed growth. Slow-release fertilizer Sustane 12-12-12 (Sustane Natural Fertilizer, Cannon Falls, MN, US) was added to seedlings, and 1–2 g of Peters' 20-20-20 (JR Peters Inc., Allentown, PA, US) was applied biweekly when watering.

### Determination of the meiotic stage

**Figure 2:**
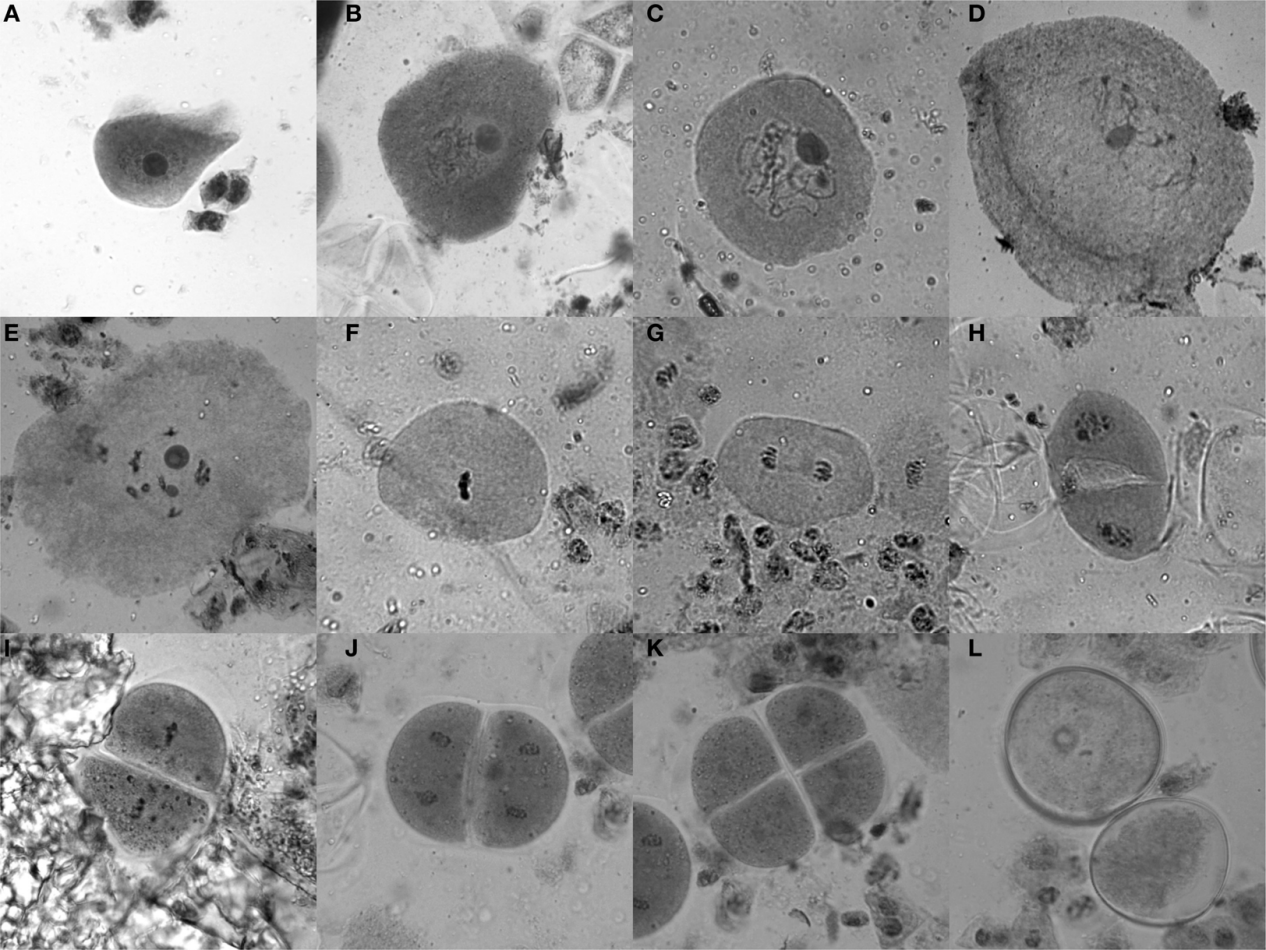
**Acetocarmine staining of maize meiotic stages. (A)** Leptotene, **(B)** Zygotene, **(C)** Pachytene, **(D)** Diplotene, **(E)** Diakinesis, **(F)** Metaphase I, **(G)** Anaphase I, **(H)** Telophase I/Interphase/Prophase II, **(I)** Metaphase II, **(J)** Anaphase II, **(K)** Tetrad stage, **(L)** Microspores.

Tassels of 6–8 week old maize plants, which are still enclosed in the stalk and located right above the last internode, usually contain anthers with cells in meiosis. To test for the stage, a vertical incision (5–10 cm) was made in the stalk to get to the tassel. Optimally, a few buds from the middle of the main tassel were taken as a sample (without harming the remaining tassel) and put into Farmer's fixative (3 parts ethanol, 1 part glacial acetic acid) (Sass, 1951), and the stalk was closed using tape as a bandage. Ten minutes in the fixative is sufficient to yield good results for the staining procedure for which anthers are dissected on a slide and stained with acetocarmine (0.5 g carmine in 55 mL water and 45 mL acetic acid, stirred while boiling for 20–30 min, filtered after cooled down). Heating the slide with the sample over an ethanol burner while stirring with an oxidized iron rod (a “rusty nail”) intensifies the stain from a bright red to dark purple. Self-made disposable pestles (plastic 200 μL pipet tips were shortly heated and thus melted at the tip, then pressed flat on a glass slide) were used to squash the anthers, keeping them in the middle of the drop. After adding a coverslip and heating once more, slides were analyzed with a bright-field microscope (Figure 2).

### Preparing the tassel for collection of precise stages

Spikelets along maize tassels occur in pairs, and both of them contain an upper and a lower floret, each containing three anthers (see simplified in Figure 1, detailed in McSteen and Hake, 2001). Meiosis progresses gradually along the tassel, thus anthers of respective florets in adjacent spikelets are usually at the same stage.

*For RNA samples*, the maize plant should be placed near the dissecting microscope. We cut or broke the stalks at the bottommost node, carried them from the greenhouse to the lab and shortened them by one more internode just before putting them into water. The bandage-tape at the tassel location was then removed, the incision lengthened if needed so the whole tassel was exposed. We marked three positions on the stalk with a pen where we took additional samples from the main tassel for checking the stage and repeated sampling and staging until the range with the desired stages was defined. For CML228, we took samples in addition from the midpoint of the smallest and the largest side tassel. The cut was closed with tape in between sampling, and for each round of collection the 10–20 upmost usable buds were removed for dissecting. *For DNA samples*, tassels can conveniently be fixed prior to collection. We removed the whole tassel from the stalk, took the section we wanted to use (usually the middle of the main tassel), and put samples from the upper and lower end and the midpoint into Farmer's fixative for further staging. The remaining tassel, dedicated to collection, was processed with methods similar to those in Pickle (2010), fixed for 10 min under vacuum (~500 mmHg) in crosslinking fixative (0.4 M sucrose, 10 mM Tris-HCl (pH 8.0), 10 mM MgCl_2_; 1% formaldehyde added immediately before use). After releasing the vacuum, 1/10 volume of 1.25 M glycine solution was added to stop the crosslinking, mixed by inverting and put under vacuum for another 5 min. After washing twice with 1× PBS, the tassels were kept in 1xPBS in the fridge till collection.

### Meiocyte isolation

#### Self-made collection device

Two glass Pasteur pipets were modified and inserted at both ends of a flexible rubber tube: For the mouthpiece, the thin part of the glass pipet was removed (forcefully broken away, which can be facilitated by first using a glass cutter at the intended break site) and the opening smoothed by heating. The glass pipet at the other end had been extended and thinned to a micro-capillary by heating the glass and then quickly stretching it out (see also Chen and Retzel, 2013). Not every self-made micro-capillary piece is optimal for collection—(i) the diameter of the opening should be almost the same or only slightly bigger than the diameter of the cell clusters, (ii) the length of the micro-capillary part of the piece should be = 3 cm, and (iii) the micro-capillary opening smooth and appearing like a perfect or only slightly misshaped ring when looking through the microscope.

#### Collection of meiocytes

Microscope slides were prepared by adding two drops of distilled water to attach two plastic coverslips (Fisherbrand, Catalog #12-547) to the slide. Three anthers from the upper florets from 10 to 20 spikelets (see Figure 1) were dissected and put into a small drop of 1× PBS (for RNA experiments with RNase inhibitors, Cat #03335399001, Roche diagnostics, Basel, Switzerland) on one of the plastic coverslips. The anthers were squashed with a disposable pestle, keeping the area covered by solution as small as possible. More 1× PBS was added onto the squashed anthers till a nice dome was formed (~50–200 μL). Under an inverted microscope (40–100×), the collection device was then used with gentle mouth-pipetting to suck the meiocyte clusters into the microcapillary (see Movie 1). Most anther wall debris floats on top of the drop while the meiocytes usually sink down to the bottom (see schematic drawing in Chen and Retzel, 2013). A purification step was performed by blowing the collected material onto a second plastic coverslip, adding 1× PBS to achieve a good dome and then repeating the collection procedure. The capillary collection tube should be emptied frequently in between collection; if it was filled with too much fluid, more meiocytes clung to the glass pipet wall. *For RNA samples:* Since collection for RNA is done on still thriving plants, we collect only for a few hours; otherwise meiosis might have progressed too far beyond the stage seen in the staining samples. After the purification step, collected meiocytes were directly put into microtube with 500 μL RNA*later*® (Ambion) or similar RNA storage solution. The collected meiocytes were stored in the fridge for 1–4 weeks, adding more collected meiocytes into the same microtubeover time. However, RNA*later*® should not be diluted to less than 0.5×; only before RNA extraction, was the meiocyte solution further diluted with 1× PBS, mixed well, and centrifuged down (e.g., 8000 rpm, 2 min) to remove the viscous liquid with the supernatant. One additional wash with 1 mL 1× PBS left a small pellet of meiocytes that was processed immediately by adding lysis buffer as the first step of the RNA extraction. *For DNA samples:* Collected meiocytes from fixed tassels were put into −70°C at the end of the day, after pelleting (3000 rpm, 2 min) and removing most of the supernatant. When enough meiocytes were collected, they were thawed and pooled together.

### RNA extraction

In general, we followed the instructions of the RNA extraction kit (RNAqueous Micro Kit, Ambion, Catalog #AM1931), which is suited for small amounts of plant samples. Depending on the experiment, total RNA or only mRNA can be extracted, by adjusting the ethanol amount in the RNAqueous Micro Kit. We extracted total RNA and processed the sample later for making either only an mRNA library or separate libraries for both mRNA and small RNA. To grind the cells (which was done without liquid nitrogen) we used microtube pestles which are convenient to avoid loss of material. Yield was measured using the Qubit RNA BR Assay Kit (Invitrogen, Catalog #Q10210) with the Qubit Fluorometer (Invitrogen).

### RNA-Seq and small RNA-Seq

#### Sequencing libraries

*RNA-Seq:* For the genome-wide analyses of expression patterns in B73 and Mo17 inbred lines, cDNA was generated using a routine RNA library preparation TruSeq protocol developed by Illumina Technologies (San Diego, CA) using 1 μg of total RNA as input. Two replicates each of meiocytes were sampled for RNA-seq experiments from B73 and Mo17 lines. Using the kit, mRNA was first isolated from total RNA by performing a polyA selection step, followed by construction of single-end sequencing libraries with an insert size of 150 bp. Briefly, polyA selected RNA was cleaved as per Illumina protocol and the cleaved fragments were used to generate first strand cDNA using SuperScript III reverse transcriptase and random hexamers. Subsequently second strand cDNA was synthesized with RNaseH and DNA polymerase enzyme. Adapter ligation and end repair steps followed second strand synthesis. Resulting products were amplified via PCR and cDNA libraries were then purified and validated using the Bioanalyzer 2100 (Agilent Technologies). Single-end sequencing was performed on maize meiocyte samples using the Illumina HiSeq 2000 platform. Samples were multiplexed with unique six-mer barcodes and run on multiple lanes to obtain 1 × 50 bp reads. *Small RNA-Seq:* The small RNA library was prepared according to the Illumina TruSeq Small RNA preparation guide developed by Illumina Technologies (San Diego, CA) using 1 μg of total RNA as starting material. Quality of total RNA was tested using the Agilent Technologies Bioanalyzer 2100. Then, 5′ (GUUCAGAGUUCUACAGUCCGACGAUC) and 3′ (TGGAATTCTCGGGTGCCAAGG) adapters were sequentially ligated to the sample. Illumina adapters are designed such that they preferentially ligate small RNA. Ligation was followed by reverse transcription whereby cDNA fragments were generated, flanked by adapter molecules on both ends. PCR was performed using specific primers that anneal to the ends of the adapter sequences. The resulting PCR products were then size selected by polyacrylamide gel electrophoresis at 145–160 base pairs, which captures small RNA molecules up to 36 nucleotides. Resulting libraries were purified appropriately and validated using Bioanalyzer 2100. Single-end sequencing was performed on B73 meiocytes using Illumina HiSeq 2000 platform. Each sample was multiplexed (along with other samples) with unique six-mer barcodes and run on a single flow cell (7 lanes) to obtain 1 × 36 bp reads.

#### Read processing

**Figure 3:**
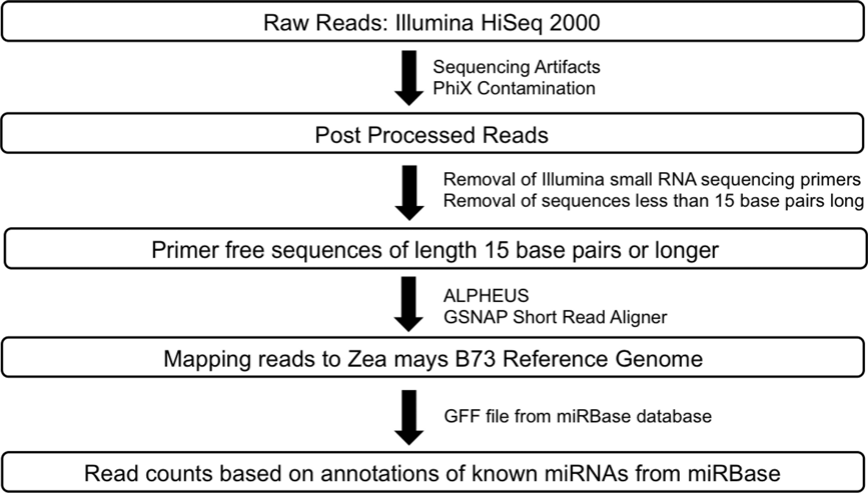
**Workflow for smRNA sequence data processing**.

*RNA-Seq:* Raw sequence data for *Zea mays* B73 and Mo17 meiocytes (2 replicates) generated by Illumina HiSeq 2000 sequencer were passed through a post processing pipeline where raw sequence data were filtered for Illumina adapters/primers and possible PhiX contamination. The post-processed reads for each of the samples were mapped back to the *Zea mays* B73 reference genome using GSNAP (**G**enomic **S**hort-read **N**ucleotide **A**lignment **P**rogram, v 2013_05_09; Wu and Nacu, 2010). Two mismatches were allowed in the 14 base pairs seed region of the sequence reads. Read counts were generated using the alignments output and the pre annotated *Zea mays* reference. *Small RNA-Seq:* The library generated for *Zea mays* B73 meiocytes was sequenced using the Illumina HiSeq 2000. Raw reads were post-processed to remove Illumina adapters/primers and PhiX contamination. A total of approximately 54.4 million 36 base pairs singleton reads were generated. Following this, the reads were further processed to detect small RNA sequencing primer that got introduced during the small RNA library preparation stage. Cutadapt (Martin, 2011; v1.2.1), a tool specifically developed to remove adapters from next generation sequence data, was used for small RNA primer/adapter trimming. Sequence data resulting from cutadapt greater than or equal to 15 base pairs were retained for further mapping and read count generation. The average read length after trimming was found to be at the expected length of 21–24 base pairs. Please refer to Figure 3 for a detailed workflow. Processed reads were then aligned to the maize B73 genome reference (RefGenv2, annotation release 5b.60) with GSNAP v 2013_05_09 (Wu and Nacu, 2010) using default parameters except max-mismatches and indel-penalty were set to 2. In order to generate read counts, associated microRNA annotation for *Zea mays* was downloaded from the miRBase sequence database release version 20 (http://www.mirbase.org/ftp.shtml). Read counts were generated using the alignments and the miRBase *Zea mays* annotation.

### Chromatin extraction

After pooled meiocytes were pelleted (3000 rpm, 2 min), all further steps were adapted from Pickle (2010) as following: first adding 100 μL extraction buffer 1 [0.4 M sucrose, 10 mM Tris-HCl (pH 8.0), 10 mM MgCl_2_; 5 mM β-mercaptoethanol; and 1× protease inhibitors added immediately before use]. Meiocytes were thoroughly ground using a microtube pestle and then 900 μL extraction buffer 1 were added by rinsing along the pestle while removing it. We then incubated the sample on ice for half an hour, vortexing the tube periodically till the solution was quite homogenous. A square-inch piece of Miracloth was pre-wet with extraction buffer 2 (0.25 M sucrose, 10 mM Tris-HCl pH 8.0, 10 mM MgCl_2_, 1% Triton X-100; 5 mM β-mercaptoethanol; and 1× protease inhibitors added immediately before use), and a cut 1000 μL pipet tip used to filter the homogenous meiocyte solution through the Miracloth into a new microtube. The sample was then centrifuged (6000 rpm, 20 min, 4°C), the supernatant discarded, and the pellet resuspended in 1 mL extraction buffer 2 by pipetting. The resuspended pellet was transferred cautiously on top of 300 μL extraction buffer 3 (1.7 M sucrose, 10 mM Tris-HCl pH 8.0, 2 mM MgCl_2_, 0.15% Triton X-100; 5 mM β-mercaptoethanol; and 1× protease inhibitors added immediately before use), pre-loaded in a new microtube. Again the sample was centrifuged (13,000 rpm, 60 min, 4°C), the supernatant discarded, and the pellet consisting of isolated nuclei resuspended in 50 μL lysis buffer (for ChIP experiments, preferably from Magnify ChIP kit, Invitrogen, or: 50 mM Tris-HCl pH 8.0, 10 mM EDTA, 0.4% SDS; 0.1 mM PMSF, and/ or 1× protease inhibitors added immediately before use) or 50 μL TE buffer (for bisulfite conversion, 10 mM Tris, 1 mM EDTA).

### Chromatin immunoprecipitation (ChIP)

In general, we followed the instructions in the MAGnify ChIP Kit manual (MAGnify Chromatin Immunoprecipitation System, Invitrogen, Catalog #49-2024), adapted by the following specific modifications for plants and meiocyte-procedure:

#### Chromatin fragmentation

This crucial step can only be done with a sonicator adapted to handle small volume samples, i.e., without a metal probe that has to be immersed into the sample. The small volume of 50 μL made it imperative to use a sonicator bath like the Bioruptor 200-UCD from Diagenode where no direct contact between the sample and the sonication device is needed (see Results and Discussion). The conditions had to be extensively tested and optimized till the fragmentation was consistently in the range of 100–300 bp long DNA molecules. In our case, we used 90 cycles, each 30 s on, 30 s off at the high setting, the lower part of the tube submerged in a cycling cooling water bath. Centrifuging the sample down and thoroughly resuspending it with a pipet in the beginning and at least once in between supported homogenous fragmentation. Then, we proceeded with centrifuging and transferring the supernatant containing the sample as described in the MAGnify ChIP Kit manual.

#### Chromatin diluting and binding

Even though the MAGnify ChIP Kit is meant for a low number of cells per IP reaction, it assumes that the starting amount of cells and with it the cell concentration is high (1 million cells/50 μL). We adapted it to a starting concentration of 20,000 cells/50 μL by adding 400 μL dilution buffer to the whole sample. We then used 40 μL as an Input control and followed the kit manual except for a modification in the chromatin binding where we first incubated 200 μL of diluted chromatin extract with the beads 6 h (or overnight), replaced it with 200 μL of the remaining chromatin extract for another 6 h (or overnight) incubation instead of incubating 100 μL once for 2 h.

#### Crosslinking reversal and DNA purification

For the 40 μL we used as Input control, we scaled up and thus added 172 μL Reverse Crosslinking Buffer + 1 μL Proteinase K. When eluting the DNA after the purification, we used 50 μL Elution Buffer instead of 150 μL to get a higher concentration that is measurable. We still had to measure 5 or even 10 μL to be in a range detectable by fluorometry with the Qubit dsDNA High Sensitivity Assay Kit (Invitrogen, Catalog # Q32851).

### ChIP-seq

#### Chip-seq library preparation

An Ovation Ultralow Library system from NuGen (San Carlos, CA) was used to generate libraries for ChIP-seq sequencing. Meiocyte DNA (immunoprecipitated and input control) from B73 and Mo17 inbred lines were processed for sequencing. Initial quality control with Agilent Bioanalyzer 2100 showed peaks at approximately 100–300 bp for the samples. The workflow was comprised of the following main steps: Fragmented genomic DNA was processed by end repair to generate blunt ends. Adapter ligation was then performed by an inline multiplexing (4-mer barcodes) method for B73 meiocytes. For Mo17 meiocytes, on the other hand, ligation by direct read multiplexing method (6-mer barcodes) was employed. Ligated products were amplified by PCR and purified for sequencing. Single end sequencing was performed on all multiplexed B73 and Mo17 samples using the Illumina HiSeq 2000 platform to obtain 1 × 100 bp reads.

#### Read processing and analysis

Similar to RNA-seq and Small RNA-seq analysis, the raw data from the Illumina sequencers were post-processed for sequencing artifacts, adapters, and primers and also for PhiX contamination. The post-processed reads for meiocytes in the inbred lines B73 and Mo17 were then mapped back to the maize B73 genome reference (RefGenv2, annotation release 5b.60) with GSNAP v 2013_05_09 (Wu and Nacu, 2010) with default parameters except max-mismatches = 2, indel-penalty = 2, novelsplicing = 1, localsplicedist = 1000, distantsplicepenalty = 1000, terminal-penalty = 1000; and known splice sites were fed into the alignments. The alignments were generated using a pipeline developed at the National Center for Genome Resources (Miller et al., 2008). The resulting alignments for each sample from both lines were then fed into CHANCE (**CH**ip-seq **AN**alytics and **C**onfidence **E**stimation; Diaz et al., 2012) for further quality and enrichment analysis.

### DNA bisulfite conversion

#### Pretreatment of chromatin from fixed samples

To remove any residual RNA, we added 1 μL RNAse A (10 mg/mL), incubating at 37°C for at least 1 h. For de-crosslinking, we added 1 μL Proteinase K (20 mg/mL) and incubated at 60°C for 2–6 h. The DNA was then precipitated by adding 1/10th volume of 3 M sodium acetate (5 μL) and 3 volumes cold 100% ethanol (150 μL), mixing thoroughly by pipetting or flicking and inverting the tube, and putting the sample into −70°C for overnight or longer. After centrifuging at full speed for 30 min at 4°C (13,000 rpm in a tabletop microfuge), the supernatant was removed, 500 μL cold 70% ethanol added, mixed, and centrifuged again (15 min, 4°C, 13,000 rpm). After removing the supernatant, the pellet was allowed to dry for 1 h or till all liquid was gone, then dissolved in 50 μL TE buffer. The DNA amount was measured using 1 μL pure or further diluted DNA solution with the Qubit dsDNA High Sensitivity Assay Kit with the Qubit Fluorometer. In addition, an agarose mini gel with ~5 μL was run to check for sample degradation.

#### DNA bisulfite conversion and library preparation

The B73 meiocyte DNA sample was processed with the bisulfite library preparation protocol recommended by Bio Scientific (Austin, TX). Genomic DNA was fragmented to the desired size (200–350 bp) and an end repair step was then performed on the samples to generate blunt ends. This was followed by 3′ adenylation to facilitate the process of ligation with methylated adapters. It is during this step that the bisulfite-seq barcodes (Illumina sequencing compatible) are attached to the inserts. Purified ligated DNA products (at 250–300 bp) were subjected to the bisulfite conversion step using the EZ DNA Methylation-Gold Kit (Zymo Research Corp, Cat #D5005). In this reaction, non-methylated cytosines are converted to uracil (read as thymine when sequenced) using heat (98°C) and the conversion reagent. The conversion step was followed by PCR amplification, purification, and library validation. Libraries were then sequenced on the Illumina HiSeq 2000 platform to generate 2 × 100 bp paired-end data.

#### Read processing and analysis

Raw 2 × 100 bp data from Illumina HiSeq 2000 was post-processed for sequencing artifacts, adapters, and primers and for PhiX contamination. Meiocyte samples were sequenced at 4.5× genome coverage. Data was analyzed as per EpiGnome Methyl-Seq Bioinformatics User Guide (rev.0.1). This user guide incorporates other open-source software packages like Bismark, Bowtie (Langmead et al., 2009; http://bowtie-bio.sourceforge.net/index.shtml), Trimmomatic, and SAMtools (Li et al., 2009). V0.30 of the command line tool Trimmomatic (Lohse et al., 2012), was used to trim and crop Illumina fastq poor quality data as well as to remove adapters. This program was run on paired-end mode using recommended parameters. Using Bismark Bisulfite Mapper (Krueger and Andrews, 2011; user guide v0.8.3), a bisulfite converted genome was first prepared for *Zea may*s reference (RefGen_v2). This was followed by aligning the paired-end data to the converted *Zea mays* genome. Finally, using bismark_methylation_extractor, methylation call was extracted for every cytosine (C) analyzed. The position of every C was reported depending on its context, i.e., CpG, CHG, CHH, or other.

## Results and discussion

### Meiocyte isolation of different inbred lines

**Figure 4:**
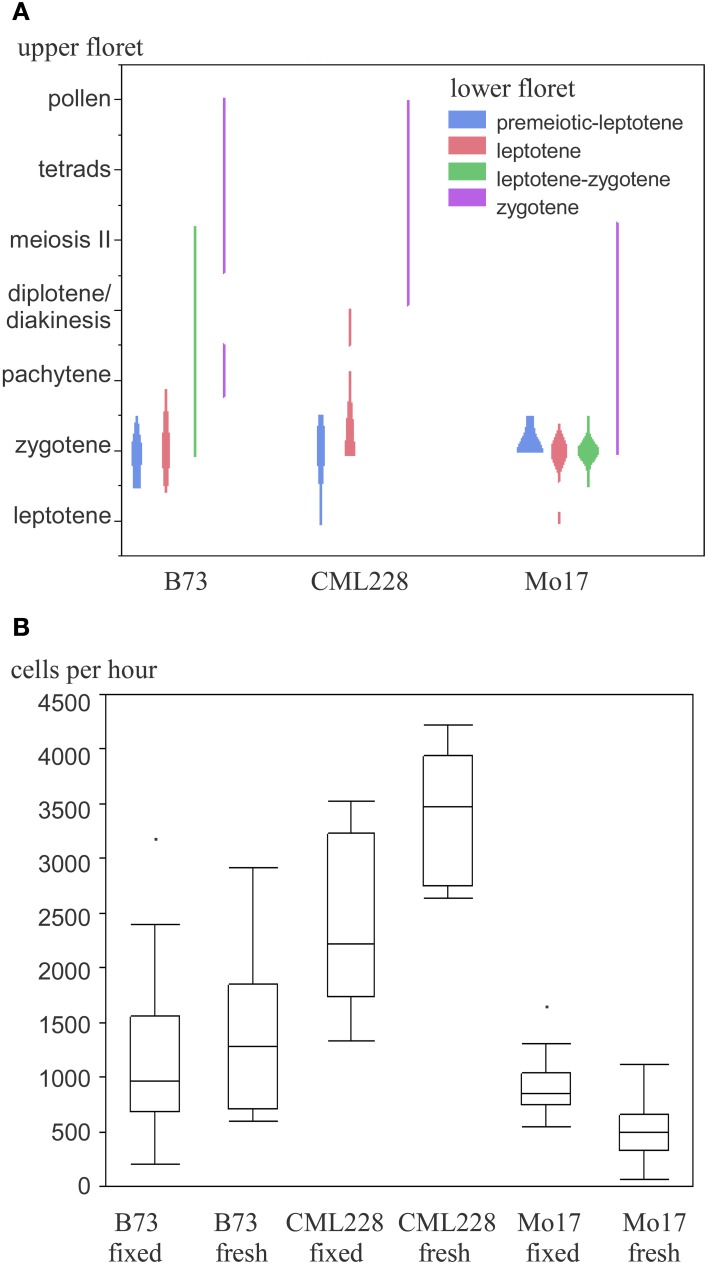
**Maize meiotic stages and meiocyte collection efficiency of different inbred lines. (A)** Occurrence of paired meiotic stages in upper and lower florets. **(B)** Collection efficiency in fixed and fresh material.

We used the maize inbred lines B73, Mo17, and CML228 in our studies, whose genomes are structurally diverse (Liu et al., 2003). For B73, which is the most commonly used inbred line for research, both mutant lines and an assembled reference genome are available (Schnable et al., 2009). Mo17 is of interest since it is a parent, with B73, for a widely used hybrid with a pronounced heterosis effect. CML228 is a tropical line and of interest to researchers due to its different recombination characteristics. Different inbred lines from maize have notable differences with respect to their tassel growth, meiotic progression timing, and synchrony along the tassel and between main and side tassels. Maize tassels have a main branch and several smaller lateral branches. Spikelets occur in pairs and each has two florets—an upper larger one and a lower smaller one (see simplified in Figure 1, detailed in McSteen and Hake, 2001). In general, the developmental timing differs along the tassel as well as between the florets of a spikelet. The meiotic stage in lower florets usually was found to be 1 day behind the stage in upper florets in B73, Mo17, and CML228 (Figure 4A).

While B73 had meiocyte clusters where some cells had often already dissociated during early prophase I, CML228 had bigger and more intact clusters. The amount of collected meiocytes was estimated and finally more accurately determined. For an approximation, we counted the prophase clusters collected in the purification step. Since many clusters were broken in pieces or had cells dissociated, we used 15 cells per cluster as a rule-of-thumb. For intact clusters, especially CML228 ones, we counted single clusters as 2 or 3 to be closer to the actual number of meiocytes contained. The amount of cells could be determined in a more exact way by taking two samples of a well-mixed meiocyte solution (e.g., 20 μL out of 500 μL), counting individual cells, and using the average to calculate the cell amount in the whole volume (e.g., 25 times as many). We experienced a significant difference between hybrid lines regarding collection efficiency (Figure 4B). Most cells per hour (up to 4200) could be collected using CML228; both B73 and CML228 could vary highly in collection efficiency, while Mo17 had a far smaller range of cells collectable per hour, and also the lowest average (Figure 4B).

Other observations we made during meiocyte collections with impact on quality and quantity of collected cells concern fixation, the surface used for the collection drop, and tapetal cells still attached to meiocyte clusters. To achieve the best fixation, it was crucial to insert the tassel in e.g., a Falcon tube in the normal direction (top at top), and tap the tube lightly till most air bubbles clinging to the buds were gone. To keep the tassel completely submerged, we added a piece of Miracloth on top, and a sufficient fixative volume was used (at least 5 times more than the tassel is occupying). In collection drops for maize meiocytes, a lot of starch granules could be seen at the bottom, which we avoided and diluted by applying a purification collection step (Movie 1). In rare cases, and differing between hybrids and stages, tapetal cells were still attached to the meiocyte cluster, and the whole clusters were then avoided when isolating pure meiocytes. Crucial for the microcapillary collection method is a high dome of the collection drop, so anther wall parts can float on the surface, far apart from the opening of the collecting microcapillary which is guided along the bottom of the drop. We found that plastic coverslips do a better job than expensive ProbeOn slides in allowing the PBS form a drop with a high dome.

### RNA studies

We started this suite of maize experiments with RNA-seq for transcriptome analysis, following up on a successfully performed equivalent study for *Arabidopsis* from our lab (Chen et al., 2010). We started with ~40,000 isolated meiocytes, decreasing the amount in further experiments, and can now report that as few as ~5000 meiocytes can be used without compromising yield or coverage. RNA-seq library protocols use ~1 μg of total RNA, which is about one quarter of our usual RNA yield (Table 1). We do not see a linear correlation between cell amount used and RNA yield, which might be attributed to varying efficiency in breaking up all cells in the initial grinding step and fluctuations in correct estimation of cell number and RNA measuring. Using different methods for RNA quantification can give results that vary two-fold or more, both between platforms like Nanodrop, Agilent Bioanalyzer, and Qubit, and between dilutions or different amounts used on the same platform. The Qubit system turned out to be the most reliable one in our studies.

**Table 1:** **RNA yield**.

**Material**	**Amount**	**Total RNA (μg)**	**Illumina reads**	**Reads aligning**	**Unique reads**
B73 meiocytes	~40,000 cells	4.6	35,158,570	31,561,071 (89.8%)	28,965,646 (82.4%)
B73 meiocytes	~40,000 cells	6.4	77,551,649	68,789,860 (88.7%)	63,201,742 (81.5%)
B73 meiocytes	~20,000 cells	7.94	36,703,499	18,997,952 (51.8%)	13,761,384 (37.5%)
Mo17 meiocytes	~5000 cells	2.64	19,474,524	16,331,942 (83.9%)	14,899,276 (76.5%)
Mo17 meiocytes	~10,000 cells	6.28	15,529,520	12,738,246 (82%)	11,531,212 (74.3%)
CML meiocytes	~20,000 cells	3.97		Experiment in progress	

Small RNA studies were performed with ~20,000 isolated meiocytes which yielded sufficient RNA for small RNA library generation. Approximately 1 ug of total RNA was used for library preparation. Sequence reads generated were of high quality with average Phred score that was greater than 35 as validated by FastQC, a quality control software for high throughput sequence data (Babraham Bioinformatics Institute). A total of 54.4 million 36 bp single-end reads were generated which corresponded to approximately 1× genome coverage. After adapter trimming and eliminating short sequences (<15 bp), 36.7 million (888,965,189 bp) high quality reads were retained. This further reduced genome coverage to 0.4×. Alignment statistics indicated that 52% (38% unique) of the reads mapped back to the reference.

### Chromatin extraction

When using more input material for chromatin extraction, like for seedlings as a control, we noticed that DNA degradation can occur sometimes when dissolving in TE buffer at the last step of the chromatin extraction. Lysis buffer could be used instead, which contains SDS that inhibits DNase. The RNase incubation step as part of the bisulfite conversion pre-treatment was then extended and might not have been as efficient. Alternatively, DNA degrading activity in seedlings might have stemmed from an earlier step when frozen seedling material thawed before extraction buffer 1 was added for chromatin extraction.

### ChIP

**Figure 5:**
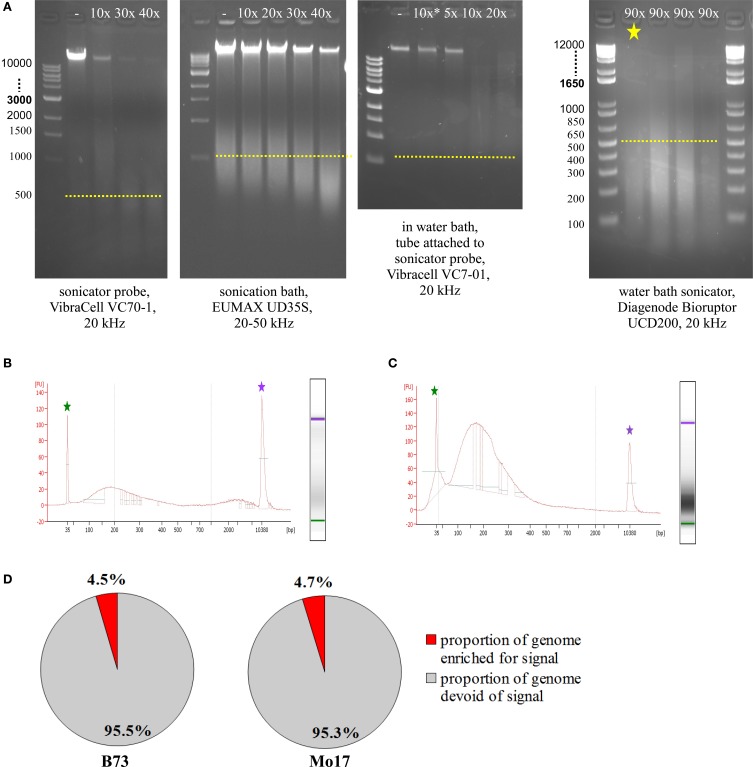
**Sonication optimization and ChIP enrichment. (A)** Agarose gel electrophoresis of samples treated with different sonicators. **(B)** Agilent Bioanalyzer analysis of sample sonicated with the Bioruptor, Diagenode (same sample as in **A**, marked by yellow asterisk). **(C)** Agilent Bioanalyzer analysis of sample used for ChIP, sonicated with the Bioruptor, Diagenode. **(D)** Genome enrichment in H3K4me3-ChIP of meiocyte samples.

Prior to running test ChIPs, sonication had to be optimized, so that DNA was consistently fragmented into pieces of 100–300 bp length. To check fragmentation, we used 5–50 μL of the sample, de-crosslinked overnight at 60–65°C, added 1 μL RNase A (10 mg/mL), incubated 1 h at 30°C, added 1 μL Proteinase K (20 mg/mL), and incubated at least 1 h at 60–65°C. We then loaded most of the sample on an agarose gel or used 1 μL on an Agilent Bioanalyzer DNA High Sensitivity Chip. The most commonly used sonicating devices consist of an ultrasound-emitting probe which has to be inserted into the sample solution. Unfortunately, this is not a very feasible approach for small amounts of cells in just 50 μL volume. Scaling up the volume to 150–200 μL makes it possible to use a sonicator probe which can yield appropriate fragmentation with 40 cycles (Figure 5A, VibraCell). Scaled-up volume however makes the downstream analysis more challenging, we even had to adjust the Magnify ChIP Kit to cope with 50 μL of sonicated cells; approaches to concentrate higher starting volumes were not satisfactory, either due to loss of material or to unfavorable conditions for downstream processing. Sonication baths are better suited for small amounts of volume, since no probe has to be inserted, but the whole tube containing the sample can be immersed into a water bath with ultrasound transmitted throughout. The first sonication bath tested yielded some fragmentation with 40 cycles but did not do so reproducibly and also left a substantial part of the genomic DNA completely un-fragmented (Figure 5A, EUMAX sonication bath). A combination, using the VibraCell sonicator probe in a water bath with the whole tube attached to the probe, resulted in clear fragmentation but with fragments not small and focused enough and also hard to reproduce, even with higher cycle number than shown (Figure 5A, EUMAX probe in water bath). Choosing to use the Bioruptor UCD200 sonication bath (Diagenode) which is specifically designed for highly reproducible and gentle DNA fragmentation finally gave the desired results (Figure 5A, Bioruptor sonicator). When checking sonication efficiency with Agilent Bioanalyzer High Sensitivity Assay (Figure 5B), fragment size concentrated from 100–300 bp, with an additional but smaller increase of fragments above 2000 bp. We noticed that the upper peak can be avoided by resuspending the sample thoroughly before and once or twice during the 90 cycles (30 s on, 30 s off) of sonication at the level high (Figure 5C). Occasionally, samples were not adequately fragmented, which results in low genomic enrichment when analyzing the sequencing data. CHANCE (Diaz et al., 2012) was used to perform quality control steps and evaluate if samples exhibited enrichment. Among other reports, CHANCE generates a summary statement describing the statistical significance of immunoprecipitation enrichment (or the lack thereof). It also generates pie-charts estimating the percentage of the genome enriched for biological signal which helps in evaluation if the experiment was successful (Figure 5D).

The ChIP procedure as described in Materials and Methods can be performed with diverse antibodies, but each might need testing and optimizing of conditions. We successfully used the rabbit polyclonal anti-trimethyl-histone (Lys4) antibody from Millipore (Catalog #07-473). A first ChIP test run recovered most DNA when 10 μL antibody were used, and further tests showed that yield is higher when the incubation step time is extended (Table 2). The DNA amount recovered in our ChIP experiments still varied (0.1–11.7% of the starting amount) but gave sufficient Illumina reads for downstream analysis (Table 3, Figure 5D).

**Table 2:** **Optimizing antibody incubation**.

**Material**	**AB incubation**	**Starting amount DNA (ng)**	**ChIP yield (ng)**	**ChIP yield (%)**
B73 anthers	1 μL AB, 2 × 2 h	100	0.01	0.0
B73 anthers	5 μL AB, 2 × 2 h	100	0.17	0.2
B73 anthers	**10μL AB**, 2 × 2 h	100	**6.13**	**6.1**
B73 anthers	IgG control, 2 × 2 h	100	0.16	0.2
B73 seedlings	10 μL AB, 2 × 2 h	250	7.56	3.0
B73 seedlings	10 μL AB, **2 × >6 h**	250	**23.76**	**9.5**
B73 anthers	10 μL AB, 2 × 2 h	300	0.32	0.1
B73 anthers	10 μL AB, **2 × >6 h**	300	**22.50**	**7.5**

**Table 3:** **ChIP yield**.

**Material**	**Starting amount DNA (ng)**	**ChIP yield (ng)**	**ChIP yield (%)**	**Illumina reads**	**Reads aligning**	**Unique reads**
B73 seedlings	200	3.67	1.8	47,721,479	39,338,146 (82.4%)	35,936,314 (75.3%)
B73 meiocytes	50	2.11	4.2	54,594,613	46,856,597 (85.8%)	43,374,762 (79.4%)
B73 meiocytes	~100	10.45	10.5	140,601,359	108,612,489 (77.2%)	91,570,391 (65.1%)
B73 anthers	~1000	117	11.7	62,729,940	47,144,419 (75.2%)	39,015,601 (62.2%)
B73 seedlings	450	3.17	0.7	75,233,218	60,523,843 (80.4%)	53,321,841 (70.9%)
Mo17 anthers	~1700	1.42	0.1	74,214,399	38,453,885 (51.8%)	29,646,577 (39.9%)
Mo17 meiocytes	440	0.3	0.1	66,290,033	50,204,574 (75.7%)	44,673,223 (67.4%)

### DNA methylation

DNA methylation studies were performed on isolated meiocytes similar to the other experiments described in this study. Paired-end sequencing yielded 108,406,934 total reads with genome coverage of ~4.5×. Average quality of raw reads was found to be greater than 35 as determined by FastQC. Trimming reads via Trimmomatic did not reduce the data by much (108,350,928). Methylation analysis indicated 47% alignment rate and reported methylated C's in different contexts (Table 4) suggesting that the experimental design and conversion step of the protocol were successful. The percentage of methylated cytosine in different contexts did not differ substantially from that of seedlings (Table 4). We are now focusing on detailed analysis of the locations where DNA methylation differs between meiocytes and seedlings, seeking to extend findings connecting DNA methylation with gene expression (Furner and Matzke, 2011; Gent et al., 2013). The DNA methylation data obtained from meiocytes will also be used to examine how DNA methylation correlates with recombination, which has been suggested in different organisms (Sigurdsson et al., 2009; Melamed-Bessudo and Levy, 2012; Mirouze et al., 2012).

**Table 4:** **Detected DNA methylation**.

**Cytosine context**	**Total**	**Methylated**	**Unmethylated**	**Percentage methylated (vs. seedlings) (%)**
CpG	144,076,576	123,259,971	20,816,605	85.6 (vs. 85.4)
CHG	144,535,224	99,913,963	44,621,261	69.1 (vs. 68.9)
CHH	610,639,792	11,668,133	598,971,659	1.9 (vs. 1.8)
Unknown (CN or CHN)	93,854	5434	88,420	5.8 (vs. 5.9)

## Conclusions

The described methods have been established and successfully applied in our labs. In the case of RNA-seq, our previous study done in *Arabidopsis* (Chen et al., 2010) and a more recent one done in maize (Dukowic-Schulze et al., 2013) showed that it is imperative to use isolated meiocytes to gain new insight into the specific events occurring in meiosis. Although data from whole anthers can give an approximate view of the meiotic transcriptome, transcripts from anther wall cells dilute and influence the final result. Especially for DNA-based approaches, we strongly recommend against using whole anthers, especially when looking at ubiquitous features like DNA methylation or also small RNA, where we found significant differences between anthers and meiocytes.

## Author contributions

Stefanie Dukowic-Schulze performed lab experiments, Anitha Sundararajan and Thiruvarangan Ramaraj conducted most data analysis. Stefanie Dukowic-Schulze and Anitha Sundararajan wrote the manuscript. Changbin Chen and Joann Mudge designed the original research. All authors edited the manuscript, and approved the final version.

### Conflict of interest statement

The authors declare that the research was conducted in the absence of any commercial or financial relationships that could be construed as a potential conflict of interest.

## Supplementary material

Movie 1 **Capillary collection of meiocytes**.
